# Advances in the application of extracellular vesicles derived from three-dimensional culture of stem cells

**DOI:** 10.1186/s12951-024-02455-y

**Published:** 2024-05-01

**Authors:** Wenya Chen, Peipei Wu, Can Jin, Yinjie Chen, Chong Li, Hui Qian

**Affiliations:** 1 Department of Orthopaedics, Affiliated Kunshan Hospital of Jiangsu University, Kunshan, 215300 Jiangsu China; 2 Jiangsu Key Laboratory of Medical Science and Laboratory Medicine, Department of Laboratory Medicine, School of Medicine, Jiangsu University, 301 Xuefu Road, Zhenjiang, 212013 Jiangsu China; 3 Department of Laboratory Medicine, The First Affiliated Hospital of USTC, Division of Life Sciences and Medicine, University of Science and Technology of China, Hefei, 230001 Anhui China

**Keywords:** Stem cells, Extracellular vesicles, 3D cell culture, Clinical applications, Therapeutics

## Abstract

Stem cells (SCs) have been used therapeutically for decades, yet their applications are limited by factors such as the risk of immune rejection and potential tumorigenicity. Extracellular vesicles (EVs), a key paracrine component of stem cell potency, overcome the drawbacks of stem cell applications as a cell-free therapeutic agent and play an important role in treating various diseases. However, EVs derived from two-dimensional (2D) planar culture of SCs have low yield and face challenges in large-scale production, which hinders the clinical translation of EVs. Three-dimensional (3D) culture, given its ability to more realistically simulate the in vivo environment, can not only expand SCs in large quantities, but also improve the yield and activity of EVs, changing the content of EVs and improving their therapeutic effects. In this review, we briefly describe the advantages of EVs and EV-related clinical applications, provide an overview of 3D cell culture, and finally focus on specific applications and future perspectives of EVs derived from 3D culture of different SCs.

**Graphical Abstract:**

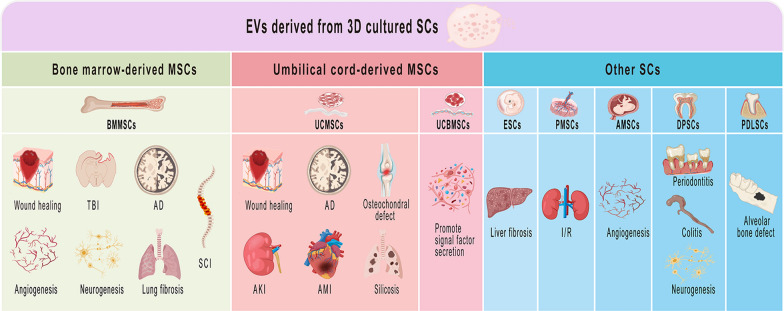

## Introduction

Stem cells (SCs) are a class of cells with unique capacity for self-renewal and multi-lineage differentiation [1]. There are various different types of SCs based on their organizational origin: bone marrow-derived SCs, umbilical cord-derived SCs, adipose tissue-derived SCs (AdSCs), embryo-derived SCs (ESCs), and many others [2]. According to their differentiation potential, they can be classified as totipotent SCs, pluripotent SCs, multipotent SCs, oligopotent SCs and unipotent SCs [3]. Given the intrinsic characteristics of SCs, their therapeutic effects have been evaluated in preclinical studies or clinical trials in neurodegenerative diseases, cardiovascular diseases, genetic disorders, and oncology [4]. With the further development of stem cell research, nevertheless, researchers have found that SCs may lead to the formation of tumors and disease transmission when applied, and that immune rejection, stringent preservation and transport conditions further limit the application of SCs [5–7]. For these reasons, stem cell-derived extracellular vesicles (EVs) with low toxicity and low immunogenicity [8] have attracted the attention of researchers.

EVs are lipid bilayer particles secreted by cells, including exosomes (Exos), ectosomes, oncosomes, microvesicles (MVs), microparticles, apoptotic bodies, and other subtypes [9]. According to their size EVs can be categorized into “small EVs” (< 200 nm) and “medium/large EVs” (> 200 nm) [10]. Exosomes are structures with an average size of 100 nm [11] and MVs range typically from 150 to 1000 nm [12]. EVs can be produced by almost all cell types, and be extracted from diverse tissues, cell culture supernatants and body fluids, which contain various bioactive molecules such as proteins, lipids, nucleic acids, growth factors, and cytokines [13, 14]. EVs, as important mediators of intercellular communication, can deliver their contents by binding to surface receptors, fusion with cells, endocytosis, phagocytosis, or macropinocytosis, thus affecting the physiological and pathological functions of the organism [15, 16]. SCs exert therapeutic effects mainly through their paracrine functions, and EVs are important effectors of their paracrine effects [17]. Numerous experimental studies showed that EVs derived from SCs (SC-EVs), especially mesenchymal stromal cells (MSCs), also known as mesenchymal stem cells [18], played a significant role in the treatment of respiratory diseases [19, 20], kidney diseases [21–23], liver diseases [24, 25], neurological diseases [26], cardiovascular diseases [27], and other diseases. As a cell-free component, EVs have high structural and compositional stability, and the ability to target injured cells, overcoming the shortcomings of stem cell therapy [8]. It is a potential alternative to stem cell therapy and a promising next-generation treatment.

However, the applications of EVs present significant challenges of low yields and lack of a proven biomanufacturing platform for efficient production [28]. The traditional two-dimensional (2D) planar culture of SCs for obtaining EVs requires enormous manpower, material and financial resources. Furthermore, the 2D planar cell culture cannot ideally simulate the physiological microenvironment in vivo, which forces changes in the cell behavior and biological functions, thus compromising the obtained EVs [29–31]. In addition, it has been shown that EVs derived from 2D planar culture of SCs (2D-EVs) have limited yield and efficacy [32, 33]. In order to overcome the limitations of 2D cell culture, three-dimensional (3D) cell culture emerged as the times require. 3D cell culture means that cells are cultured in a 3D environment that more realistically resembles the microenvironment in vivo, so that the cell–cell and cell-extracellular matrix can thoroughly interact with each other, and the morphology, behavior and function of cells can be closer to natural conditions [34–37]. EVs derived from 3D cultured SCs (3D-EVs) have high yield, improved activity and varied contents [32, 38–40], at the same time, researchers have demonstrated that 3D-EVs are significantly better than 2D-EVs in treating different disease models such as kidney injury [41], liver fibrosis [42], spinal cord injury (SCI) [43], periodontitis [44], and other diseases.

Therefore, we focus on the applications of 3D-EVs. In this review, we firstly discuss the advantages of EVs applications, explaining why researchers switch from stem cell research to EVs research. Then, we briefly describe the applications of 2D-EVs and the existing clinical studies related to EVs. Next, we introduce the history of 3D cell culture and the existing 3D cell culture technologies. Finally, we concentrate on exploring the characteristics of 3D cultured SCs and its EVs, and the existing research progress, in order to lay the foundation for EVs clinical transformation.

## Advantages of EVs applications

There are many limitations to the development of stem cell research, so researchers are increasingly focusing on their replacement components, EVs. EVs from different cell sources mediate different biological functions and exhibit similar abilities to those of the cells from which they are derived [45]. SC-EVs show similar effects to SCs in disease treatment and tissue regeneration [45]. A study on lung injury showed that MSC-derived EVs (MSC-EVs) were as effective as MSCs in protecting against hyperoxic injury in newborn rats [46]. Additionally, EVs have their own unique advantages.

As a cell-free therapy, EVs can avoid the risk of immune rejection and tumor formation [8]. EVs are simpler than their stem cell sources, and they have low immunogenicity due to their lower content in membrane-bound proteins [47]. EVs do not produce endotoxin and are characterized by low cytotoxicity [48]. The cell-free structure of EVs and their inability to replicate help to prevent potential tumorigenic actions [49]. Meanwhile, due to the small size of EVs, intravenous infusion delivery of EVs also decreases the risk of vascular obstruction [50]. In addition, EVs have certain targeting properties. It has been demonstrated that intravenously injected MSC-EVs can be detected in damaged organs 1 h after application and remain in damaged tissues up to 7 days after administration [50]. Moreover, a study on MSC-EVs in renal ischemia–reperfusion (I/R) injury also showed that EVs were able to accumulate in the injured kidney. The renal function of mice in the intravenous EVs group was largely alleviated, whereas mice in the PBS group had severely damaged kidneys, which also indicated the possibility of EVs transport to damaged kidneys via the peripheral circulation pathway, suggesting a certain targeting of EVs [51]. Xu et al. also confirmed that aerosolized inhaled EVs mainly accumulated in the lungs of chronic asthmatic mice, and effectively attenuated their allergic airway inflammation and remodeling [52]. EVs are free to enter and exit the central nervous system (CNS) under both physiological and pathological conditions [53]. Studies on the applications of EVs to CNS diseases have shown that EVs can cross the blood–brain barrier, a complex biological barrier, to achieve therapeutic effects [54]. And it has been shown that MSC-EVs attenuate blood–brain barrier disruption in ischemic stroke model mice [55]. Stability is also one of the advantages of EVs. In a suitable storage solution, EVs can be stored at − 80 °C for 6 months and can maintain their original biological activity [56]. Due to the properties of natural molecular transport and good biocompatibility [57], EVs can be used as delivery carriers to carry therapeutic molecules to enhance their targeting and therapeutic effects. For example, researchers used peptide CAQK-modified, siRNA-loaded EVs for SCI therapy, and engineered EVs promoted targeted repair of traumatic SCI [58]. As a carrier, EVs can also be used in the treatment of cancer, liver disease, kidney disease, immune system disease and others [59]. Compared with the current cell therapy, EVs may achieve the same therapeutic efficacy as the source cells and have almost negligible risk of tumor formation and immune rejection, with lower storage and transport requirements, so EVs are more suitable for clinical treatment than SCs therapy.

## Clinical studies of stem cell-derived EVs

Considering the drawbacks of stem cell therapy, EVs, as important paracrine action of cells, have received increasing attention from researchers. In this section we briefly describe the preclinical studies on EVs derived from 2D cultured SCs and the clinical applications of EVs currently available.

EVs are attracting attention as a new cell-free therapeutic agent, and SC-EVs, especially MSC-EVs, have innate therapeutic potential. Ridzuan et al. demonstrated that EVs derived from 2D cultured human umbilical cord MSCs (hucMSCs) significantly attenuated bronchial and perivascular inflammation and ameliorated alveolar septal loss in cigarette smoke-induced chronic obstructive pulmonary disease (COPD) rats [60]. Human umbilical cord MSC-derived EVs (hucMSC-EVs) also alleviated neuronal damage in rat spinal cord and promoted the repair of SCI [61]. Meanwhile, EVs derived from 2D cultured hucMSCs also showed protective effects in liver I/R injury [62]. EVs derived from 2D cultured human bone marrow MSCs (hBMMSCs) were able to modulate brain immune responses induced by focal brain injury and reduce neuroinflammation [63]. Another study showed that human bone marrow MSC-derived EVs (hBMMSC-EVs) were efficacious in mitigating the progression of unilateral ureteral obstruction-induced renal fibrosis in rats, displaying an anti-fibrosis effect [64]. In addition, in a glutamate-induced rat retinal injury model, adipose MSC-derived EVs were shown to reduce retinal excitotoxicity and markedly improve the morphological and functional abnormalities of the inner retinal layer [65]. Moreover, iPSC-derived EVs (iPSC-EVs) could promote wound repair in diabetic mice through anti-inflammatory immunomodulatory effects [66]. Compared with the natural application of SC-EVs, it is also possible to pre-treat the parental cells to obtain EVs with corresponding properties or to engineer EVs for drug loading and surface modification to obtain better therapeutic effects [67].

The therapeutic effects of SC-EVs have been demonstrated in many preclinical experiments, and clinical researchers are gradually applying EVs to clinical trials (Tables 1 and 2). Among the listed studies on the clinical applications of EVs, there are not only evaluations of their therapeutic effects, but also assessments of the safety and tolerability of EVs from various kinds of cells. It may be the application of a certain SC-EVs alone [68] or the combination of EVs with the underlying method of treating the disease [69]. For ongoing clinical trials (Table 1), EVs derived from BMMSCs and umbilical cord MSCs (UCMSCs) are the most used, and a minority of EVs derived from iPSCs. The researchers have intervened different diseases by intravenous injection, local injury site injection, inhalation, dressing and drops in COVID-19, acute respiratory distress syndrome (ARDS), perianal fistula, anti-aging, burns, liver failure and other diseases, which shows that EVs are applied in various ways in clinical trials, and the research is being carried out with great enthusiasm, and EVs play an important role in clinical translation. Clinical researchers have begun to gradually apply EVs to patients with diseases, which indicates that EVs have the potential to become a new therapeutic agent in clinical applications in the future.

**Table 1:** Ongoing clinical trials with SC-EVs

NO	Study title^*^	Status	Condition	Type of EV	Intervention	NTC number
1	Bone Marrow Mesenchymal Stem Cell Derived Extracellular Vesicles Infusion Treatment for ARDS	Not yet recruiting; phase 1/2	ARDS	BMMSC-EVs	Intravenous infusion	NCT05127122
2	Safety of Extracellular Vesicles for Burn Wounds	Recruiting;phase 1	Burns	BMMSC-EVs	Dressing	NCT05078385
3	A Safety Study of IV Stem Cell-derived Extracellular Vesicles (UNEX-42) in Preterm Neonates at High Risk for BPD	Terminated; phase 1	BPD	BMMSC-EVs	Intravenous infusion	NCT03857841
4	Extracellular Vesicle Infusion Treatment for COVID-19 Associated ARDS	Completed; phase 2	COVID-19, ARDS	BMMSC-EVs	Intravenous infusion	NCT04493242
5	Bone Marrow Mesenchymal Stem Cell Derived Extracellular Vesicles Infusion Treatment for Mild-to-Moderate COVID-19: A Phase II Clinical Trial	Withdrawn; phase 2	COVID-19	BMMSC-EVs	Intravenous infusion	NCT05125562
6	ExoFlo™ Infusion for Post-Acute COVID-19 and Chronic Post-COVID-19 Syndrome	Not yet recruiting; phase 1/2	COVID-19, postviral syndrome, dyspnea	BMMSC-EVs	Intravenous infusion	NCT05116761
7	Study of ExoFlo for the Treatment of Medically Refractory Ulcerative Colitis	Recruiting; phase 1	UC, IBD	BMMSC-EVs	Intravenous infusion	NCT05176366
8	A Clinical Study on Safety and Effectiveness of Mesenchymal Stem Cell Exosomes for the Treatment of COVID-19	Recruiting; early phase1	COVID-19 pneumonia	UCMSC-Exos	Nebulized inhalation	NCT05787288
9	Safety and Efficacy of Umbilical Cord Mesenchymal Stem Cell Exosomes in Treating Chronic Cough After COVID-19	Recruiting; early phase1	Long COVID-19 syndrome	UCMSC-Exos	Nebulized inhalation	NCT05808400
10	Exosome of Mesenchymal Stem Cells for Multiple Organ Dysfuntion Syndrome After Surgical Repaire of Acute Type A Aortic Dissection	Not yet recruiting; NA	Multiple organ failure	UCMSC-Exos	Intravenous infusion	NCT04356300
11	The Effect of Stem Cells and Stem Cell Exosomes on Visual Functions in Patients With Retinitis Pigmentosa	Recruiting; phase 2/3	RP	UCMSC-Exos	Subtenon injection	NCT05413148
12	Effect of Mesenchymal Stem Cells-derived Exosomes in Decompensated Liver Cirrhosis	Recruiting; phase 2	Decompensated liver cirrhosis	HucMSC-Exos	**/**	NCT05871463
13	MSC-Exos Promote Healing of MHs	Unknown; early phase1	MHs	HucMSC-Exos	Intravitreal injection	NCT03437759
14	MSC EVs in Dystrophic Epidermolysis Bullosa	Not yet recruiting; phase 1/2	Dystrophic epidermolysis bullosa	MSC-EVs	Dressing	NCT04173650
15	MSC-EV in Acute-on-Chronic Liver Failure After Liver Transplantation	Not yet recruiting; phase 1	Liver failure, acute on chronic	MSC-EVs	Injection	NCT05881668
16	Intra-articular Injection of MSC-derived Exosomes in Knee Osteoarthritis (ExoOA-1)	Not yet recruiting; phase 1	Osteoarthritis, knee	MSC-Exos	Intra-articular injection	NCT05060107
17	Safety and Tolerability Study of MSC Exosome Ointment	Completed; phase 1	Psoriasis	MSC-Exos	Ointment	NCT05523011
18	Induced Pluripotent Stem Cell Derived Exosomes for the Treatment of Atopic Dermatitis	Recruiting; early phase1	Atopic dermatitis;	iPSC-Exos	Drops	NCT05969717
19	Induced Pluripotent Stem Cell Derived Exosomes Nasal Drops for the Treatment of Refractory Focal Epilepsy	Recruiting; early phase1	Refractory focal epilepsy	iPSC-Exos	Nasal drip	NCT05886205
20	Safety of Injection of Placental Mesenchymal Stem Cell Derived Exosomes for Treatment of Resistant Perianal Fistula in Crohn's Patients	Unknown; phase 1/2	Perianal fistula in patients with CD	HPMSC-Exos	Injected in fistula tract	NCT05499156

^*^ Study titles are indicated as listed on ClinicalTrials. https://clinicaltrials.gov/.Accessed 25 September 2023

**Table 2:** Published clinical trials with SC-EVs

**NO**	Clinical trial ID^*^	Condition	Type of EV	Cell Culture Method	Outcome	Refs.
1	/	Severe COVID-19	HBMMSC-Exos	/	17 of 24 (71%) SARS-CoV-2-positive patients recovered and 3 of 24 (13%) were stable but still in critical condition. Overall, Exos were safe and effective in treating severe COVID-19	[71]
2	ChiCTR2000030261	COVID‑19 pneumonia	HucMSC-Exos	2D cell flask culture	Nebulization therapy with Exos did promote the absorption of pulmonary lesions and decrease the duration of hospitalization in patients with mild cases of COVID-19 pneumonia without causing allergic reactions	[72]
3	/	Refractory perianal fistula in IBD	HucMSC-Exos	2D cell flask culture	The results within 6 months of receiving Exos therapy showed that 4 patients responded to treatment, 3 patients demonstrated complete healing, 1 patient showed no improvement, and none of the 5 patients experienced adverse effects	[73]
4	/	CKD	Human UCBMSC-EVs	2D monolayer culture	Human UCBMSC-EVs improved the inflammatory immune response and enhanced overall renal function in patients with grade III-IV and were safe for patients	[68]
5	NCT05402748	Complex perianal fistula	HPMSC-Exos	2D cell flask culture	Among 11 patients with complex perianal fistulae, 1 patient did not improve significantly and 5 patient’s tracts recovered completely. All patients had no allergic reactions or complications	[74]
6	/	COPD	HPMSC-Exos	2D multiflask culture	Exo-d-MAPPS treatment significantly enhanced pulmonary status and quality of life in COPD patients, and none of the 30 COPD patients experienced adverse effects, and Exo-d-MAPPS was well tolerated	[75]
7	/	Acne scars	Human AdSC-Exos	/	Fractional CO2 laser combined with Exos treatment for acne scars resulted in a greater reduction in the percentage of scars clinically assessed, less erythema, and a shorter post-treatment recovery time with fewer side effects	[69]
8	NCT04276987	Severe COVID-19	Human adipose MSC-Exos	2D cell-factory culture	Seven patients with severe COVID-19 pneumonia treated with nebulized Exos had varying degrees of regression of pulmonary lesions, which was more obvious in four of them. All patients tolerated the Exos nebulization well	[76]
9	NCT04313647	Healthy volunteers	Human adipose MSC-EVs	2D cell-factory culture	Twenty-four healthy volunteers tolerated EVs nebulization well, with no serious adverse events during the 7-day observation period	[77]

^*^Clinical trial ID is from ClinicalTrials. https://clinicaltrials.gov/ and Chinese Clinical Trial Registry. https://www.chictr.org.cn/. Accessed 25 September 2023

Among the nine published clinical trials on EVs (Table 2), eight trials showed that EVs play a therapeutic role in the corresponding diseases, while only one was an evaluation of the safety of EVs in healthy patients, which showed that EVs were well tolerated by healthy volunteers and had no adverse events occurred during the observation time, suggesting that EVs can perform a curative effect in the patients and are safe for the human beings. Seven of these clinical trials indicated that EVs they used were derived from 2D cultured SCs, while the other two did not indicate the culture method. Clinical experiments require extensive EVs, while the amount of EVs obtained from 2D cultured SCs is limited. Additionally, the growth of cells on 2D culture vessels with adherent walls, unlike the 3D environment of cells in vivo, may be restricted, resulting in altered cell–cell and cell-extracellular matrix interactions, which may prevent the cells from fully mimicking the biological functions in vivo, and the characteristics of the resulting EVs may be compromised [40, 70]. Therefore, increasing number of researchers are focusing on EVs derived from 3D cultured SCs.

## 3D cell culture

In this section, we aim to gain a preliminary understanding of 3D cell culture by introducing the origin, development history, and existing technologies. The main focus will be on 3D cell culture technologies, which can be categorized into two main types: scaffold-free technologies (including forced floating, hanging drop, magnetic levitation, and stirring spinner flask culture) and scaffold-based technologies (comprising natural scaffolds, synthetic scaffolds, and hybrid scaffolds).

### The history of 3D cell culture

Exploration of 3D cell culture can be traced back to the early twentieth century. In 1907, Wilson et al. made the first observation of biological recombination by demonstrating that siliceous sponges degenerated and dissociated into many small cell clumps when preserved in limited confinement. These cells were found to have the ability to self-organize and regenerate, eventually forming complete sponges [78]. Until decades later, Moscona A and Moscona H conducted experiments using limb-bud and mesonephric cells from chicken embryos, proving that these early embryonic organ rudiments can be rebuilt in vitro to form aggregates and restore their characteristic tissue-type development even after complete dissociation [79]. Ehrmann et al. successfully cultured different human cell lines with collagen derived from rat tails, resulting in the formation of cell mass aggregates without the use of scaffolds in 1956 [80]. In 1978, Haji-Karim et al. demonstrated the formation of spherical aggregates of different tumor cell lines by culturing them on plastic surfaces covered with liquid inert substrate [81]. In the late 1980s, isolated intestinal cells could form intestinal-like organoids in vitro through the action of collagen gel was confirmed by Montgomery [82]. The development of 3D cell culture further advanced with the successful isolation and cultivation of human embryonic stem cells (hESCs) derived from blastocysts in 1998 [83]. Levenberg et al. showed that static 3D polymer scaffold culture could promote the growth, differentiation and formation of three-dimensional vascular networks in hESCs in 2003 [84]. Subsequent studies demonstrated that AdSCs cultured in 3D dynamic spinner flasks exhibited better morphology, viability and greater differentiation capacity compared to cells cultured statically [85]. Dynamic 3D culture could enhance the properties and therapeutic potential of MSCs [86]. From no scaffolds to the use of gels and scaffolds, and from static to dynamic culture, 3D cell culture has gradually provided a more favorable environment for cell growth. With the progress of biomaterials science, the emergence of 3D cell culture based on microcarriers [87], 3D printed scaffolds [88], hollow fiber bioreactors [28], and other methods have provided new directions for the development of 3D cell culture.

### Available 3D cell culture technologies

**Fig. 1:**
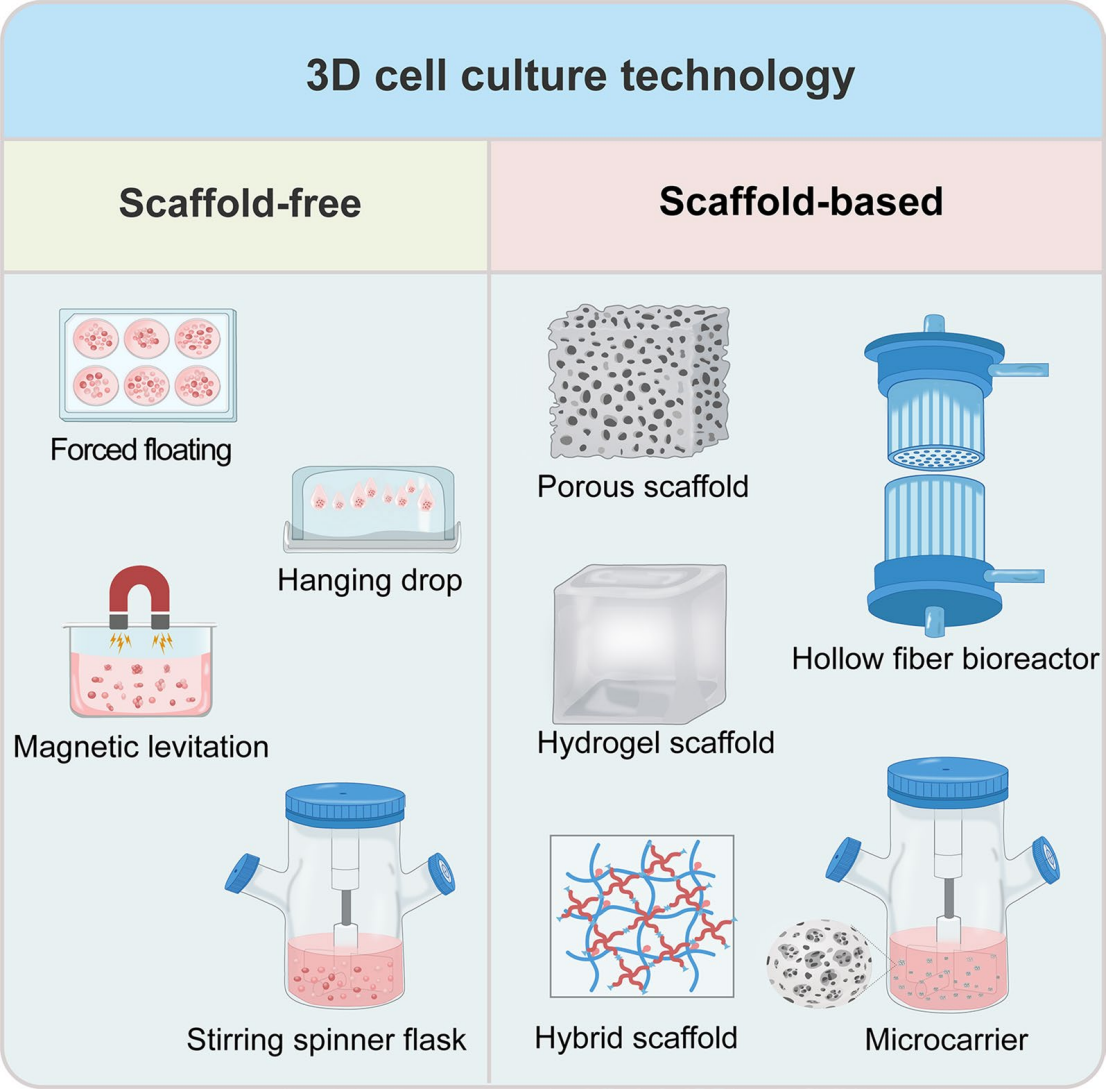
Available 3D cell culture technologies. They are mainly divided into scaffold-free and scaffold-based technologies. Scaffold-free technologies include forced floating (ultra-low attachment plate), hanging drop, magnetic levitation and stirring spinner flask. Scaffold-based technologies include porous and hydrogel scaffolds made from natural or synthetic materials, hybrid scaffolds, hollow fiber bioreactors and microcarrier-based bioreactors

At present, there are a variety of 3D cell culture technologies, which can be mainly divided into two categories: scaffold-free technologies and scaffold-based technologies (Fig. 1).

Scaffold-free 3D cell culture is where cells self-assemble to form spheroids in the absence of scaffolds [89]. The major ones include forced floating, hanging drop, magnetic levitation and stirring spinner flask culture. The forced floating method primarily uses ultra-low attachment plates, which are usually treated with hydrophilic or hydrophobic coatings, or agarose, to prevent cell adhesion to the substrate, promoting spheroid formation [90, 91]. Although this method is simple and easy to execute, it cannot control the number of cells per spheroid and is not easy to repeat [89]. The hanging drop method eliminates surface attachment by placing cell suspensions in droplets at the underside of petri dish lids, where gravity induces cell aggregation at the bottom of the droplets to form spheroids [92, 93]. While this method offers good repeatability, it is complicated to change the culture medium and apply to cell-based assays [91]. In the magnetic levitation method, cells are incubated with nanoparticles to make them magnetic overnight, then resuspended and re-seeded, and a magnetic field is applied to aggregate cells at the gas–liquid interface and form aggregates [94]. This method can rapidly form spheroids and is applicable to a variety of cell types [95]. However, all of these methods have a common issue: the culture medium is in a stagnant state, which can result in uneven nutrient supplies and improper waste disposal, thus adversely affecting the long-term culture of the spheroids. Therefore, the stirring-based dynamic spinner flask culture without scaffold is more suitable for the long-term culture of spheroids. In the spinner flask, the impeller provides sufficient nutrient supplies by continuously stirring the medium to uniformly suspend the cells, while reducing the adhesion of the cells to the solid surface and increasing the cell-to-cell contact, thereby facilitating spheroid formation [96].

Scaffold-based 3D cell culture uses scaffolds to simulate extracellular matrix components, providing structural and mechanical support for cell culture [70], as well as promoting cell attachment and growth [97]. These scaffolds can be classified into three types: natural scaffolds, synthetic scaffolds and hybrid scaffolds [98]. Natural scaffolds mainly include scaffolds made of natural biomaterials such as collagen [99], fibrin [100, 101], gelatin [102, 103], hyaluronic acid [104], chitosan [105], alginate [106], agarose [107], matrigel [108] and other biomaterials. On the other hand, synthetic scaffolds contain scaffolds synthesized from materials such as polyethylene glycol (PEG) [109, 110], polyvinyl alcohol (PVA) [111], poly lactic-co-glycolic acid (PLGA) [112, 113], polylactic acid (PLA) [114], polycaprolactone (PCL) [115, 116] and others [70]. Both natural and synthetic materials can be fabricated into porous scaffolds for 3D cell culture [105, 112]. While natural scaffolds offer better biocompatibility, they may vary between batches or donors. In contrast, synthetic scaffolds possess stronger mechanical properties and better control over scaffold characteristics, allowing adjustments based on specific requirements to address batch variations, but they lack bioactivity [91, 117]. Hybrid scaffolds, combining raw materials from both natural and synthetic scaffolds, aim to overcome these limitations of both [118]. For example, hybrid scaffolds made from synthetic PEG and natural collagen have been demonstrated to have sufficient mechanical strength and higher cell adhesion to further enhance the efficiency of adipogenic differentiation of adipose-derived SCs [110]. The alginate-PVA scaffold, produced through a solvent casting method, has been demonstrated that it can promote the wound healing efficiently and quickly [119]. Furthermore, natural hydrogels or synthetic hydrogels made from basic raw materials have important applications in 3D cultures [120–123] and the basic scaffolds can be modified with bioactive molecules to produce better scaffolds for 3D cultures [113, 124]. Hollow fiber bioreactor and microcarrier-based bioreactor cultures have also been developed on the basis of scaffolds [125]. In addition to the methods described above, cell sheet engineering [126], organ-on-a-chip [127], organoid [128], and 3D bioprinting [129] can also be used for 3D cell cultures.

## Current applications of EVs derived from 3D cultured SCs

In this section, we first introduce the characteristics of 3D cultured SCs and their derived EVs. Then we thoroughly discuss the existing applications of EVs derived from 3D cultured various sources of SCs, including bone marrow, umbilical cord, placenta, embryo, amniotic, dental pulp and periodontal ligament.

### Characteristics of 3D cultured SCs and their EVs

Growing evidences that 3D culture can better simulate the in vivo microenvironment. The biological behavior of cells in 3D culture diverges from that of cells in traditional 2D culture, and 3D culture simulates cell–cell and cell-substrate interactions more realistically [43]. 3D culture has been shown to promote the proliferation and differentiation of SCs [122, 130, 131], supporting the long-term self-renewal of them [132–135]. Bioreactor-based 3D culture could enable large-scale production of SCs [136]. Furthermore, 3D culture enhances the immunomodulatory and angiogenesis ability of MSCs, strengthens their paracrine activity, and promotes the secretion of cytokines and growth factors [137, 138]. 3D culture can alter the characteristics of SCs, and EVs are secreted by them, similarly, some studies have confirmed that 3D culture will have an effect on EVs.

3D culture of SCs has been shown to enhance their ability to secrete EVs, leading to increased production of EVs [32, 139–141]. For instance, UCMSCs cultured in hollow fiber bioreactor have been found to produce 7.5-fold higher amounts of exosomes compared to those produced by 2D culture flasks [142]. Additionally, Kronstadt et al. reported that 3D printed scaffold-perfused bioreactor culture increased the yield of BMMSC-EVs by approximately 40- to 80-fold (depending on the measurement method) compared to conventional 2D cell culture [143]. The production of exosomes by hBMMSCs in 3D culture using hang-drop and poly (2-hydroxyethyl methacrylate) coating methods was also found to be approximately twofold and 2.4-fold higher, respectively, compared to 2D monolayer culture [144]. The fold increase in EVs production may be different for various 3D culture methods when culturing different SCs, but overall 3D culture does promote the secretion of EVs.

Furthermore, 3D-EVs showed superior bioactivity [143, 145]. Haraszti et al. confirmed that exosomes derived from 3D cultured UCMSCs, obtained by tangential flow filtration, exhibited a seven-fold greater ability to transfer siRNA to neurons compared to 2D-Exos obtained by differential ultracentrifugation [32]. Kim et al. also demonstrated that EVs derived from 3D cultured hucMSCs had a stronger ability to promote angiogenesis, wound healing, and their anti-inflammatory, anti-apoptosis and anti-fibrosis abilities were also stronger than those of 2D-EVs by using in vitro angiogenesis experiment, cell migration assays, fluorescence detection and flow analysis, which may be related to the 3D-EVs’ contents [140]. In addition, hBMMSC-EVs obtained from the supernatant in hollow fiber bioreactors contained lower levels of the pro-inflammatory factors IL-6 and TNF-β, and higher levels of the immunomodulatory factor IL-8 in their contents, which had great potential in treating anti-inflammatory and immunomodulatory diseases [146]. From the above, 3D-EVs were stronger than 2D-EVs in their ability to deliver siRNA, promote wound healing, and anti-inflammation.

3D culture could alter the contents of SC-EVs [147, 148]. Culturing hBMMSCs via 3D coaxial bioprinting technology resulted in significant differences in the protein content of the derived EVs compared to those from traditional 2D cultured cells. 3D-EVs exhibited a greater diversity of proteins, with 1,023 compared to 605 in 2D-EVs, including 487 unique proteins in 3D-EVs and only 69 unique proteins in 2D-EVs. KEGG analysis also revealed that the unique proteins in 3D-EVs were mainly enriched in “metabolic pathways”, “ribosomes” and “protein processing in the endoplasmic reticulum”, whereas proteins specific to 2D-EVs were mainly associated with “axon guidance”, “complement and coagulation cascades” [149]. Additionally, 3D culture also resulted in altered miRNAs in hESCs-derived Exos. Microarray analysis showed that 39 were up-regulated and 29 were down-regulated in 3D-Exos compared to 2D-Exos, and the researchers identified miR-6766- 3p by analyzing the first 11 altered miRNAs and verified its role in liver fibrosis diseases [42].

**Fig. 2:**
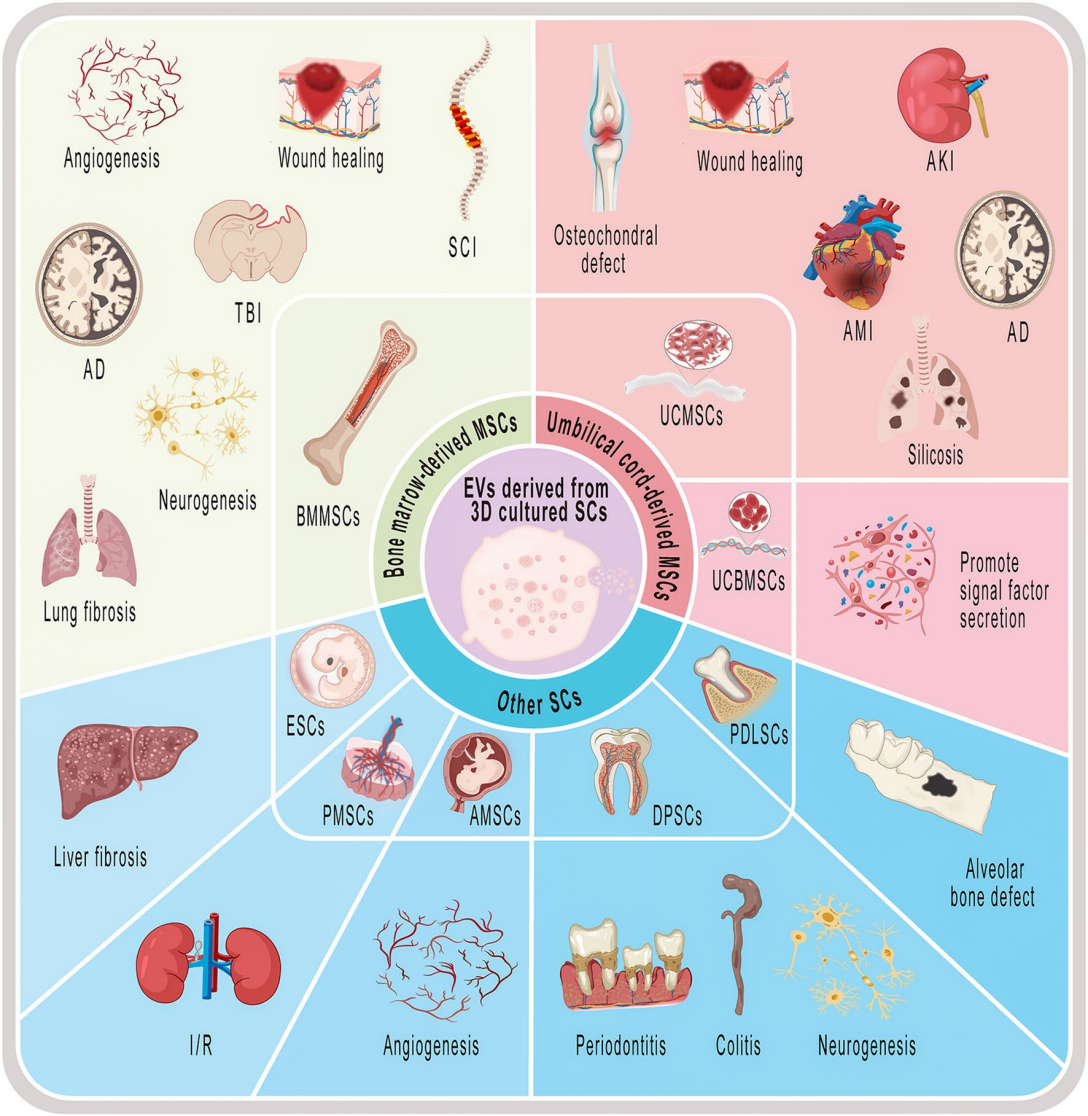
Current applications of EVs derived from 3D cultured SCs. EVs derived from 3D culture of BMMSCs for wound healing, angiogenesis, neurogenesis, SCI, TBI, AD, lung fibrosis; EVs derived from 3D culture of UCMSCs for osteochondral defect, wound healing, AKI, AD, AMI and silicosis; EVs derived from 3D culture of UCBMSCs for promoting signal factor secretion. EVs derived from 3D culture of ESCs for liver fibrosis; EVs derived from 3D culture of PMSCs for I/R; EVs derived from 3D culture of AMSCs for angiogenesis; EVs derived from 3D culture of DPSCs for periodontitis, colitis and neurogenesis; EVs derived from 3D culture of PDLSCs for alveolar bone defect

In conclusion, 3D culture of SCs enhances the production and activity of their secreted EVs while also modifying the contents of EVs, including proteins and miRNAs. This may be attributed to the influence of 3D culture on the cell shape, shear stress, extracellular matrix and other factors, which alter the characteristics of cells, thus affecting the characteristics of EVs secreted by them. It is important to note that EVs obtained from different sources of SCs using various 3D culture methods may possess distinct roles, as depicted in Fig. 2 and Table 3.

**Table 3:** Applications of EVs derived from 3D cultured SCs

No	In vivo or in vitro model	Type of EV	3D Culture method	Outcome	Refs.
	Bone marrow-derived MSCs				
1	A diabetic mouse wound healing model	HBMMSC-EVs	3D-printed scaffold perfusion bioreactor	3D-EVs promoted the percentage of wound closure and the number of neovascularization, significantly improving wound healing, whereas there was no significant difference between the vehicle control and 2D-EVs	[143]
2	In vitro wound healing model; in vitro cellular aging model	HBMMSC-EVs	3D aggregation wave reactor	Compared to 2D-EVs, 3D-EVs promoted fibroblast growth and wound healing faster and significantly reduced the number of senescent SCs, exerting anti-senescence ability	[148]
3	In vitro model for angiogenesis and neurogenesis	HBMMSC-MVs	3D culture based on PEG hydrogel microwell arrays	3D-MVs significantly stimulated HUVEC tube formation than IBE-MVs and VEGFs, but their ability to stimulate neural stem cell differentiation and proliferation was less than that of IBE-MVs, although it was stronger than that of NGFs	[159]
4	In vitro trigeminal ganglia neurite growth, elongation and complexity assays	HBMMSC-EVs	3D bioreactor based on microcarriers	Compared with 2D-EVs, 3D-EVs promoted neurite growth more, induced neurite elongation, and significantly increased neurite branching and complexity	[160]
5	A well-established controlled cortical impact rat model of TBI	HBMMSC-Exos	3D collagen scaffold culture	There was no significant difference between 2D-Exos and 3D-Exos in improving the recovery of sensorimotor function, promoting angiogenesis in rats after TBI, but 3D-Exos enhanced spatial learning better than 2D-Exos	[161]
6	5XFAD mouse model	HBMMSC-EVs	3D aggregation culture using ultra-low attachment plate	Compared with the saline group, 3D-EVs treatment significantly improved cognitive ability, reduced amyloid plaque deposition and the amount of GFAP in the brain, slowing down the progression of AD in 5XFAD mice	[162]
7	SCI rat model	SD rats BMMSC-Exos	3D culture of GelMA-based hydrogels	3D-Exos was more effective than 2D-Exos in reducing inflammation and neuroglial scarring, and promoting nerve regeneration after SCI	[43]
8	In vitro wound healing model	BMMSC-EVs	A novel microcarrier-based vertical-wheel bioreactor	The bioreactor EV group closed scratches at a faster rate than the 2D-EV group	[139]
	Umbilical cord-derived MSCs				
9	Excision wound healing mouse model	HucMSC-EVs	3D spheroid culture using an orbital shaker	3D-EVs better promoted dermal fibroblast migration, improved reepithelialization of the epithelial layer, and facilitated wound closure in excision wound model mice than 2D-EVs	[147]
10	In vivo wound healing splinting rat model	HucMSC-Exos	3D spinner flask culture	Compared with 2D-Exos, 3D-Exos had a stronger effect on reducing the area of granulation tissue in vivo, promoting complete re-epithelization of the wound, and facilitating skin healing after injury	[163]
11	A rat knee osteochondral defect model	HucMSC-Exos	3D printed acellular cartilage extracellular matrix scaffold culture	3D-Exos had enhanced ability to modulate the microenvironment of the articular cavity compared to 2D-Exos, and when used in conjunction with microporous scaffolds it could better repair osteochondral defects	[164]
12	Cisplatin-induced AKI mouse model	HucMSC-Exos	The hollow fiber bioreactor	3D-Exos are more renoprotective than 2D-Exos in ameliorating cisplatin-induced AKI	[28]
13	APP/PS1 double transgenic mice with alzheimer's disease	HucMSC-Exos	3D graphene scaffold culture	3D-Exos reduced amyloid-β production and more dramatically improved the memory and cognitive deficits in AD mice, with a stronger therapeutic effect than 2D-Exos	[165]
14	A rat model of AMI	HucMSC-EVs	The hollow fiber bioreactor	3D-EVs were able to significantly inhibit cardiomyocyte apoptosis, promote angiogenesis, and improve cardiac function in rats with acute myocardial infarction with strong cardioprotective effects compared with 2D-EVs	[33]
15	Silica-induced silicosis mouse model	HucMSC-Exos	Microcarrier-based 3D dynamic culture	3D-Exos inhibited silica-induced pulmonary fibrosis and improved lung function	[166]
16	Tube formation assay; wound healing assay; TNF-α induced inflammation model; TGF-β induced fibrosis model	HucMSC-EVs	3D spheroid culture using StemFIT 3D® plate and polydimethylsiloxane (PDMS)-coated flask	The researchers verified the pro-angiogenic, pro-wound healing, anti-inflammatory, and anti-fibrotic effects of EV- derived in 2D culture, 3D culture and 3D culture stimulated with a combination of TNF-α and IFN-γ (TI), of which TI-intervened 3D culture enhanced the mentioned functions of EVs	[140]
17	In vivo rabbit cartilage defect model	UCMSC-Exos	The hollow fiber bioreactor	Defective cartilage treated with 3D-Exos showed more neo-tissue formation and better fusion of the surrounding hyaline cartilage, which was superior to 2D-Exos in cartilage repair	[142]
18	Migration and proliferation of murine fibroblasts in vitro	UCMSC-Exos	3D spheroid culture using the aggrewell system	Exosomes derived from 3D serum-free cultured UCMSCs significantly increased migration and proliferation of murine fibroblasts, with the potential to accelerate wound healing	[167]
19	CD14 + cell migration assay, coculture of 661W cells and MVs	Human UCBMSC-MVs	3D spheroid culture using the hanging drop protocol	3D-MVs inhibited the migration of CD14 + cells better and stimulated the secretion of signaling factors from 661W cells at a stronger rate than 2D-MVs	[168]
	Other derived SCs				
20	CCL4 in conjunction with employing alcohol- induced liver fibrosis model in mice	HESC-Exos	3D spheroid culture using ultralow attachment plate	Compared with 2D-Exos, 3D-Exos significantly accumulated in the liver of fibrotic mice, significantly reduced the expression of pro-fibrotic markers and liver injury markers, inhibited liver fibrosis, and restored liver function	[42]
21	The acute renal I/R injury mouse model	HPMSC-EVs	3D spheroid culture using ultralow attachment plate	Compared with 2D-EVs, 3D-EVs were able to counteract apoptosis and inflammatory responses more effectively, resulting in reduced tissue damage, improved renal function, and better protection against I/R progression	[41]
22	In vitro HUVEC migration assay and capillary-like formation assay	HAMSC-Exos	3D spheroid culture using ultralow attachment plate	Both 2D and 3D hAMSC-Exos at the same concentration induced capillary-like formation and endothelial cell migration, and there was no significant difference between the two	[169]
23	Ligature-induced periodontitis model, DSS-induced colitis model	HDPSC-Exos	3D spheroid culture using ultralow attachment dish	Compared to 2D-Exos, 3D-Exos exerted enhanced amelioration of periodontitis and colitis	[44]
24	In vitro dorsal root ganglia neuron sprouting assay	HDPSC-EVs	3D Fibra-Cell scaffold culture	3D-EVs generated by dynamic scaffold cultured cells showed a stronger ability to stimulate neuronal axon sprouting compared to 2D-EVs	[170]
25	6-OHDA -induced apoptosis in human dopaminergic neurons	HDPSC-Exos	3D microcarrier cell culture	3D-Exos significantly inhibited 6-OHDA-induced apoptosis in dopaminergic neuronal cells, showing neuroprotective properties, whereas no such effect was observed with 2D-Exos	[171]
26	SD rat alveolar bone defect model	HPDLSC-Exos	Collagen hydrogel-assisted 3D culture system	3D-Exos effectively induced alveolar bone regeneration and expression of osteogenic proteins Runx2 and OPN in SD rats	[145]

### Applications of EVs derived from bone marrow-derived MSCs

EVs derived from planar 2D cultured BMMSCs have been shown to be effective in injury repair and regeneration, promotion of osteogenic differentiation, and anti-cancer activities [63, 64, 150–157]. However, researchers have found that EVs derived from 3D cultured BMMSCs were superior to 2D-EVs in promoting wound healing and angiogenesis. In a diabetic mouse wound-healing model, BMMSCs were cultured using a 3D-printed scaffold perfusion bioreactor, and it was discovered that their EVs significantly promoted wound healing and neoangiogenesis. In contrast, EVs derived from 2D flask cultured BMMSCs did not have this effect [143]. Similar results were observed by Yuan et al. and Jeske et al., who demonstrated that EVs derived from BMMSCs cultured in 3D aggregation wave reactor and a novel microcarrier-based vertical-wheel bioreactor were able to promote cell growth and accelerate wound closure faster than 2D-EVs in an in vitro wound healing model using primary human dermal fibroblasts [139, 148]. Another study also showed that exosomes derived from 3D printed hydroxyapatite scaffolds cultured BMMSCs were more active in promoting proliferation, migration and angiogenesis of HUVEC in the mouse model of in vivo angiogenesis compared to 2D-Exos [158].

**Fig. 3:**
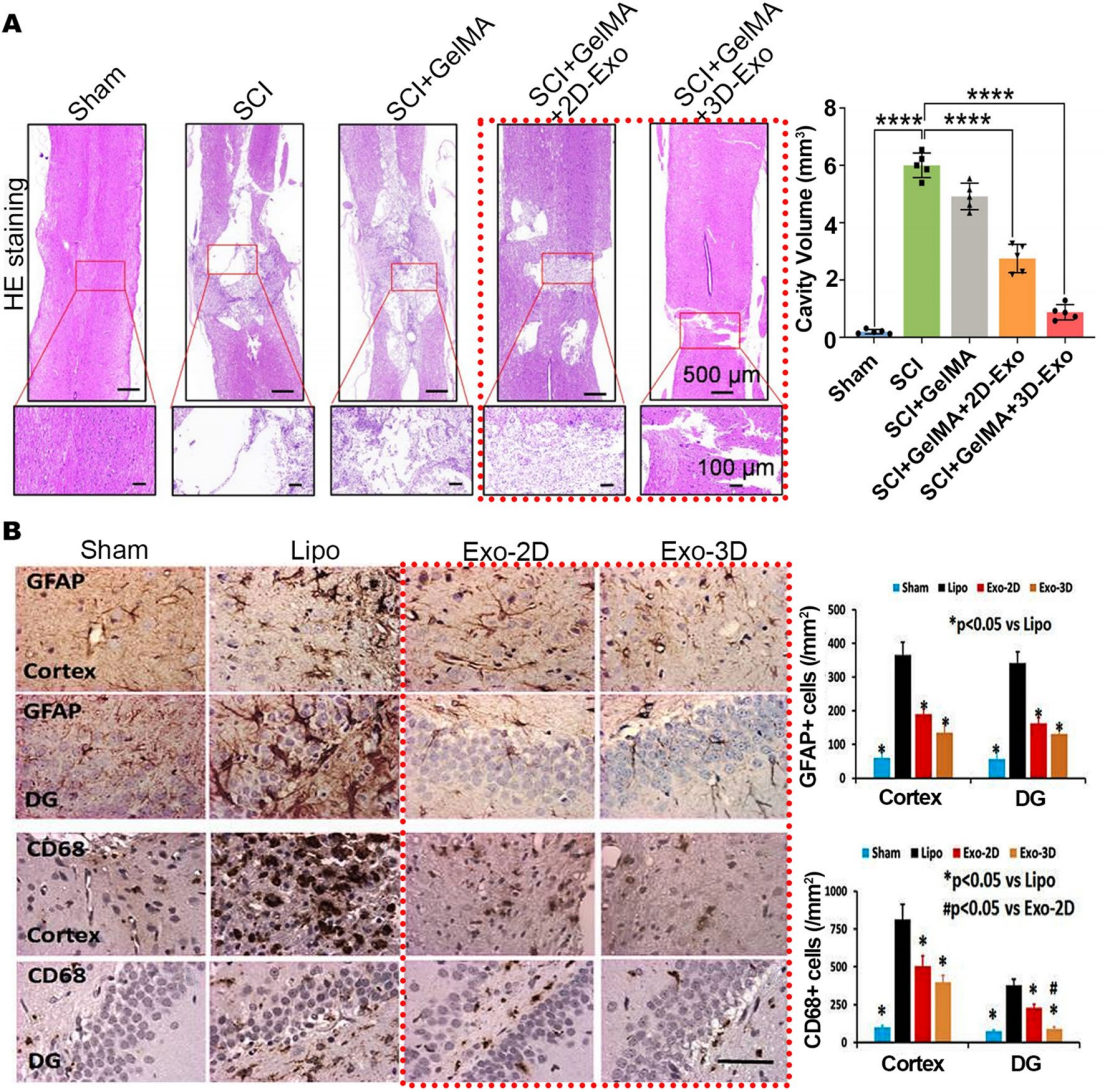
Applications of EVs derived from 3D cultured BMMSCs. **A** SD rat BMMSC-derived 3D-Exos applied to SCI rat model. Representative HE staining of tissue damage following SCI insult. Quantitative analysis of cavity volume in each group. Reproduced with permission from reference [43]. Copyright 2022, American Chemical Society **B** HBMMSC-derived 3D-Exos applied to TBI rat model. CD68 and GFAP staining and statistics on the number of positive cells in rat brain tissues. Reproduced with permission from reference [161]. Copyright 2017, Elsevier

In addition, several studies have confirmed that 3D-EVs are stronger than 2D-EVs in nerve regeneration. Han et al. established a SCI rat model, and then intervened with 2D-Exos and 3D-Exos. Exosomes derived from gelatin methacryloyl (GelMA) hydrogel hybrid 3D cultured MSCs were able to reduce the level of neuroinflammation in SCI rats, significantly decrease SCI-induced formation of cavities, improve the spinal cord tissue morphology, and attenuate SCI (Fig. 3A) [43]. Similarly, another study examined the effects of 3D-EVs derived from hBMMSCs cultured in a microcarrier-based 3D bioreactor. The researchers found that 3D-EVs were able to better promote neural neurite growth, elongation and complexity, compared to the 2D-EVs, which was demonstrated through in vitro assays using trigeminal ganglia neuronal cells [160]. Moreover, Zhang et al. obtained 3D-Exos by 3D collagen scaffold cultured hBMMSCs, and compared their effect with that of 2D-Exos in a well-established controlled cortical impact rat model of traumatic brain injury (TBI), which revealed that both of them could improve sensorimotor functional recovery and promote angiogenesis in rats after TBI without significant difference. Nevertheless, 3D-Exos strengthened the number of newborn mature neurons, significantly reduced the glial fibrillary acidic protein (GFAP) + astrocyte density and the CD68 + cell number in the damaged cortex and dentate gyrus (DG), thereby reducing brain inflammation and enhancing the spatial learning ability of the rats better than 2D-Exos (Fig. 3B) [161].In addition to promoting wound healing, angiogenesis and nerve regeneration, 3D-EVs can also reduce amyloid plaque deposition and GFAP levels in the brains of 5 familial Alzheimer’s disease (AD) mutation mice, significantly improving their cognitive abilities and slowing the progression of AD [162].

Overall, these studies suggest that EVs derived from 3D cultured BMMSCs are generally more effective than 2D-EVs in various in vitro and in vivo models. However, there are also some potential inconsistencies. For example, a study on bleomycin-induced lung injury in aged mice showed that EVs obtained by 3D spheroid culture of hBMMSCs were not as effective as 2D-EVs in terms of anti-inflammatory, anti-ECM remodeling and anti-fibrosis on lung fibrosis [172]. In conclusion, the EVs obtained from BMMSCs cultured in different 3D culture methods had different effects in different studies, some of them were similar, for example, 3D-EVs were all stronger than 2D-EVs in wound healing, but in nerve regeneration and angiogenesis the researchers would have different conclusions, and in the study of lung fibrosis the effects of 3D-EVs were not as effective as 2D-EVs. This may be due to the different responsiveness of different tissues to the contents of 3D-EVs, with some tissues being sensitive and others less responsive.

### Applications of EVs derived from umbilical cord-derived MSCs

**Fig. 4:**
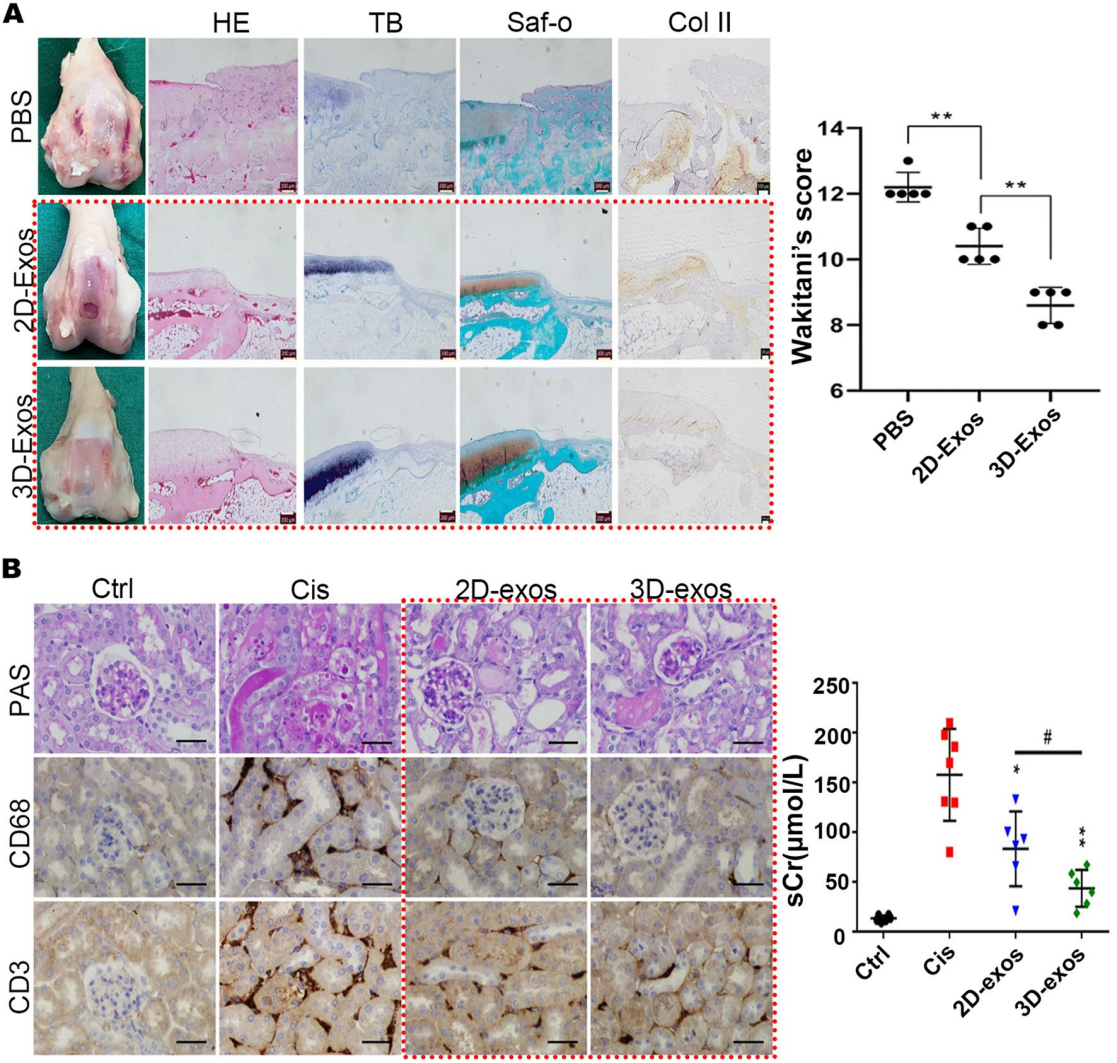
Applications of EVs derived from 3D cultured UCMSCs. **A** UCMSC-derived 3D-Exos applied to rabbit cartilage defect model. Representative macroscopic images of the regenerated tissues. Staining results of HE, TB, Saf-O and immunohistochemical staining for type II collagens. Wakitani scores for the histological sections. Reproduced with permission from reference [142]. Copyright 2020, Springer Nature **B** HucMSC-derived 3D-Exos applied to AKI mouse model. Representative images of PAS staining of renal cortex. Representative immunostaining images of CD68^+^ macrophages or CD3^+^ T cells in the tubulointerstitium. Serum creatinine graph. Reproduced with permission from reference [28]. Copyright 2020, Springer Nature

Here we present 3D culture of umbilical cord-derived SCs, mainly UCMSCs and umbilical cord blood MSCs (UCBMSCs). Similar to BMMSC-EVs, EVs derived from 2D cultured UCMSCs have been found to exert protective effects in various ways, such as acute and chronic renal injury, heart injury, liver injury, brain injury, and immunomodulation, as well as alleviation of type 2 diabetes mellitus [23, 173–179]. As with BMMSCs, in an in vivo wound healing model, EVs derived from 3D spheroid culture and 3D spinner flask culture of UCMSCs were able to promote epithelialization of wounds and accelerate post-injury skin healing, with a more dramatic effect than 2D-EVs [147, 163]. Furthermore, exosomes derived from 3D cultured UCMSCs displayed stronger effects than 2D-Exos in repairing osteochondral damage, improving osteochondral activity, and promoting cartilage regeneration. Yan and Wu et al. compared the effects of 2D-Exos and 3D-Exos in the rabbit cartilage defect model and found that 3D-Exos could increase the number of chondrocytes and enhance the function of chondrocytes by stimulating cell proliferation, migration and inhibiting apoptosis, and the effect of repairing osteochondral injury was significantly better than 2D-Exos (Fig. 4A) [142]. Another study on a rat articular osteochondral defect model also demonstrated the stronger ability of 3D-Exos to promote osteochondral repair, and the most complete osteochondral repair was achieved when 3D-Exos were used in combination with 3D-printed scaffolds [164].

EVs derived from 2D cultured hucMSCs suffered from low yield and limited therapeutic effect in the treatment of acute kidney injury (AKI) [23, 28]. Therefore, Cao et al. applied the hollow fiber bioreactor to culture hucMSCs, and the same number of hucMSCs harvested exosomes 19.4-fold higher than those harvested from cells cultured in conventional 2D culture. Furthermore, in a mouse model of cisplatin-induced AKI, treatment with 3D-Exos led to a more significant reduction in serum creatinine, IL-6, and TNF-α levels compared to 2D-Exos. Additionally, 3D-Exos exhibited a significant attenuation of proximal tubular epithelial cell vacuolar degeneration, necrosis, and cast formation, and remarkably reduced renal interstitial infiltration of inflammatory cells such as T cells (CD3 positive) and macrophages (CD68 positive), indicating a more potent therapeutic effect in attenuating cisplatin-induced AKI in mice (Fig. 4B) [28].

In addition to stronger protection against kidney injury, hucMSC-derived 3D-Exos has shown excellent results in the treatment of AD. In APP/PS1 double transgenic mice with AD, the researchers cultured hucMSCs with 3D graphene scaffold and 2D graphene film to obtain 3D-Exos and 2D-Exos, respectively. The results showed that 3D-Exos could down-regulate the β-secretase and up-regulate the α-secretase through its unique contents, thus alleviating the production of amyloid-β in AD mice, thereby more significantly improving memory and cognitive defects of AD mice. Notably, 3D-Exos exhibited stronger therapeutic effects than 2D-Exos, demonstrating its potential in the treatment of AD [165].

The role of 3D-EVs in acute myocardial infarction (AMI) has also been reported. Sun et al. verified the role of EVs derived from 2D culture and hollow-fiber-bioreactor based 3D culture of hucMSCs in a rat model of AMI by immunofluorescence staining, histological staining and echocardiographic assay. The results indicated that 3D-EVs more significantly reduced the infarcted area and fibrotic area of myocardial infarction rats, promoted angiogenesis and improved the cardiac function of rats which had a stronger cardioprotective effect [33]. In addition, the researchers validated the role of exosomes derived from 3D spinner flask culture of hucMSCs alone in a silica-induced silicosis model, without comparing it to 2D-Exos. The results showed that 3D-Exos attenuated the numbers of cellular nodules and collagen I deposition in the lungs of damaged mouse tissues, relieved the degree of interstitial fibrosis, and ameliorated the impaired respiratory function of the mice. This suggests that exosomes derived from 3D dynamic culture of husMSCs provide a new approach for improving clinical treatment induced by silica [166].

As well as the effects of EVs derived from 3D cultured UCMSCs, the researchers also studied the functions of UCBMSC-derived MVs. The results showed that MVs derived from human UCBMSCs cultured by suspension droplet method were stronger than 2D-MVs in stimulating cytokine secretion from 661W retinal photoreceptor neurons. Moreover, 3D-MVs inhibited CD14 + cell migration stronger than 2D-MVs, which might be due to the increased secretion of anti-inflammatory factors. Therefore, 3D-MVs have a better role in reducing inflammation in degenerative retinal diseases and may be a superior way to achieve regenerative therapy for retinal diseases [168].

The existing reported EVs from 3D-cultured umbilical cord-derived MSCs mainly showed stronger effects than 2D-EVs in skin wound healing, osteochondral injury repair, AKI, AD and AMI. EVs generated by umbilical cord-derived MSCs cultured using different 3D culture methods, such as spheroid culture, rotary flask culture, and hollow fiber reactor culture, all showed stronger therapeutic effects in all of the different disease models mentioned above, but their applications in other diseases still need to be explored.

### Applications of EVs derived from other SCs

**Fig. 5:**
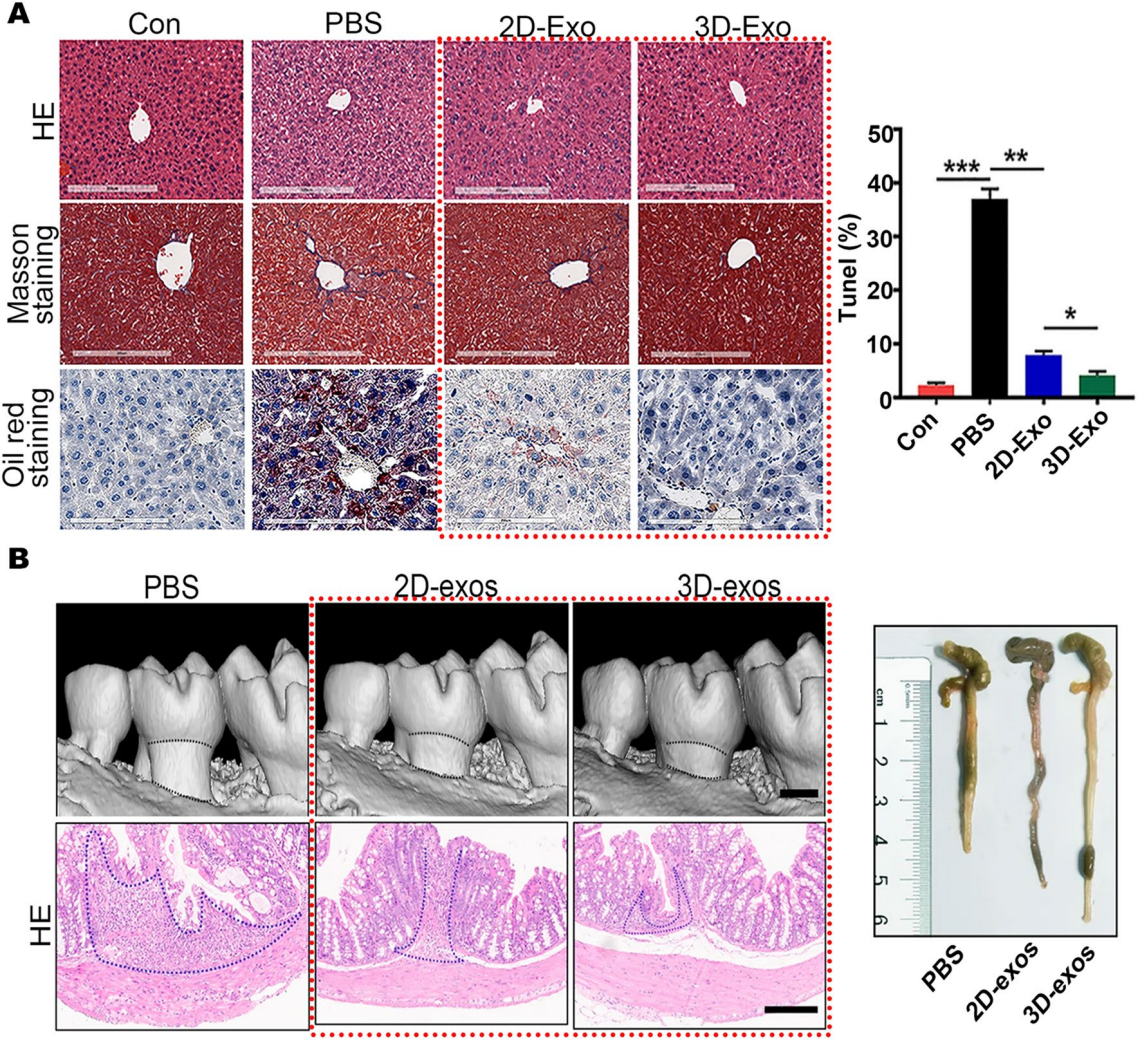
Applications of EVs derived from 3D cultured hESCs and hDPSCs. **A** HESC-derived 3D-Exos applied to liver fibrosis mice model. Representative images of HE, masson and oil red staining of liver tissue. Quantification of positive cells in TUNEL staining of liver tissues. Reproduced with permission from reference [42]. Copyright 2021, Springer Nature **B** HDPSC-derived 3D-Exos applied to periodontitis and colitis model. 3D reconstructions of maxillae revealed by micro-CT. HE staining showed histopathological changes in the colon. Representative colon pictures of the mice in each group. Reproduced with permission from reference [44]. Copyright 2021, Springer Nature

In addition to the applications of EVs derived from BMMSCs and UCMSCs, the researchers also explored the therapeutic potential of EVs derived from 3D cultured hESCs, human placental mesenchymal stem cells (hPMSCs), human amnion mesenchymal stem cells (hAMSCs), human periodontal ligament stem cells (hPDLSCs), and human dental pulp stem cells (hDPSCs). For example, Wang et al. studied EVs derived from hESCs, comparing exosomes derived from 3D spheroid cultured cells in ultra-low attachment culture plates with exosomes derived from 2D monolayer cultured cells to investigate their therapeutic effects on liver fibrosis [42]. In a mouse model of liver fibrosis induced by CCL4 and alcohol for 8 weeks, it was found that 3D-Exos significantly reduced lipid accumulation, decreased collagen I and α-SMA levels, inhibited the synthesis of pro-fibrotic markers in fibrotic livers, dramatically attenuated the expression of inflammatory markers such as IL-6, interferon-gamma (IFN-γ), and monocyte chemotactic protein 1 (MCP1) expression with enhanced anti-inflammatory effects. TUNEL results also showed that 3D-Exos significantly attenuated apoptosis in liver tissue of fibrotic mice after treatment. 3D-Exos exhibited a more pronounced inhibition of hepatic fibrosis and restoration of hepatic function than 2D-Exos, showing a significant therapeutic effect on liver fibrosis (Fig. 5A). The researchers also showed that the therapeutic effects of 3D-Exos were mediated through the targeting of the TGFβRII-SMADS pathway via its enriched miR-6766-3p [42]. Furthermore, in a renal I/R injury model, the investigators also demonstrated that EVs derived from 3D spheroid cultured hPMSCs were more effective in reducing inflammatory response, tissue damage, improving renal function and mitigating the progression of ischemic AKI compared to 2D-EVs [41]. EVs derived from 3D cultured hESCs and hPSCs showed better therapeutic effects in liver fibrosis and AKI, while in vitro HUVEC migration assay and capillary-like formation assay, no significant difference was observed between 3D-Exos derived from hAMSCs cultured in ultra-low adhesion culture plates and 2D-Exos in inducing capillary formation and endothelial cell migration [169].

For hDPSCs cultured in 3D spheroidal culture based on ultra-low adhesion petri dishes, their derived exosomes restored the balance of reactive T helper 17 cell/Treg in inflamed periodontal tissues with stronger anti-inflammatory effects and significantly reduced alveolar bone loss in a ligature-induced periodontitis model, as well as significantly reducing experimental colitis (Fig. 5B). And through miRNA sequencing, the researchers found the miRNA with the greatest difference between 3D-Exos and 2D-Exos, namely miR-1246, proving that 3D-Exos played a role in restoring the Th17cell/Treg balance through the miR-1246/Nfat5 axis, which laid the foundation for treating inflammatory bowel disease by restoring the immune response of inflamed periodontal tissue [44]. Two other researches on EVs derived from 3D cultured hDPSCs have demonstrated their neurological applications. EVs derived from 3D fibra-cell scaffolds cultured hDPSCs significantly promoted rat neuronal axon sprouting in vitro [170], while EVs derived from 3D microcarrier cultured cells showed neuroprotective properties against 6-OHDA-induced apoptosis of dopaminergic neuronal cells in vitro, making them a new potential therapeutic tool for Parkinson's disease [171]. Two studies demonstrated that 3D-EVs obtained by culturing hDPSCs in different 3D culture modalities were stronger than 2D-EVs in terms of nerve growth and protection. Similarly, exosomes derived from 3D cultured hPDLSCs exhibited stronger effects on promoting osteogenesis, proliferation, migration and inhibiting apoptosis of hBMMSCs in vitro compared to 2D-Exos. However, in vivo SD rat alveolar bone defect model, the researchers did not compare 3D-Exos with 2D-Exos, but only studied the effect of 3D-Exos. The results showed that 3D-Exos promoted the expression of osteogenic proteins Runx2 and OPN, and enhanced new bone formation in rats with alveolar bone defect [145].

These studies suggest that EVs derived from different SCs cultured in 3D methods may be more effective than 2D-EVs in preventing liver fibrosis, alleviating colitis and periodontitis, and promoting new bone formation, or may not differ, for example, in inducing angiogenesis and endothelial cell migration. Some researchers have made preliminary explorations of the mechanism of action of 3D-EVs, such as EVs derived from 3D cultured hESCs in liver fibrosis and 3D hDPSC-derived Exos in periodontitis and colitis, and some have only validated the effects of 3D-EVs. We still need further exploration of EVs derived from 3D cultured SCs.

**Fig. 6:**
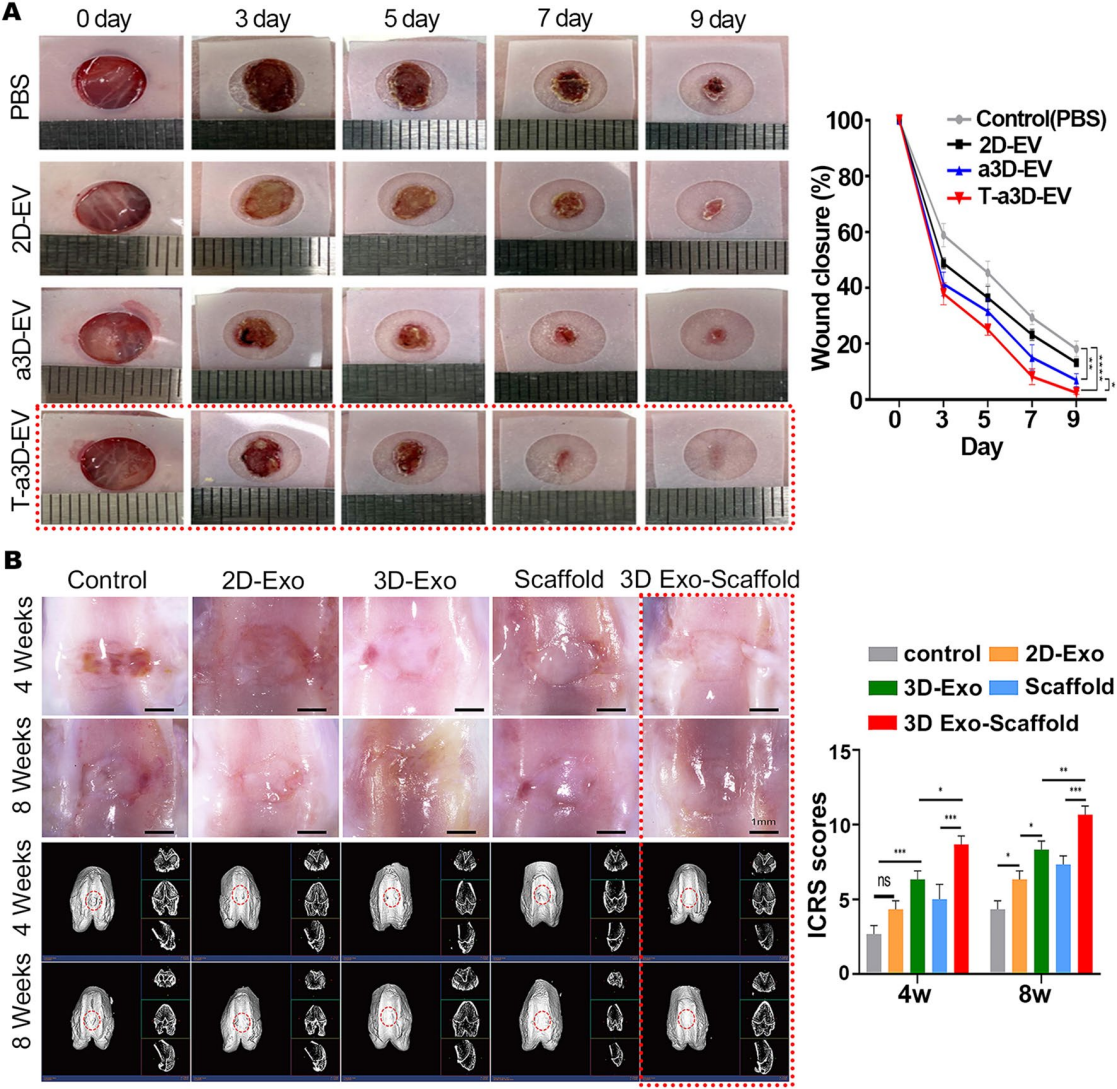
Methods to improve the efficacy of 3D-EVs. **A** HucMSC-derived T-a3D-EVs applied to wound healing mouse model. Representative in vivo wound closing images. Quantitative wound closure rate. Reproduced with permission from reference [147]. Copyright 2023, Elsevier **B** HucMSC-derived 3D-Exos combined scaffolds applied to rat knee osteochondral defect model. Microscopic observation of the repaired tissues. Micro-CT images showing 2D and 3D reconstructions of the repaired cartilage. ICRS score of the cartilage defect. Reproduced with permission from reference [164]. Copyright 2023, Elsevier

Although many studies have confirmed that 3D-EVs alone are better than 2D-EVs in injury repair and regeneration, the effects of 3D-EVs are further enhanced when they are used in combination with other experimental techniques. For example, Min Lim et al. demonstrated that when exogenous transforming growth factor beta-3 (TGF-β3) was used to interfere with an advanced 3D culture system of hucMSCs to obtain EVs (T-a3D-EVs), it was able to further increase the yield of EVs and lead to significantly improved migration of dermal fibroblasts and wound closure in an excisional wound model (Fig. 6A) [147]. 3D-Exos loaded with A151 ODN (an exogenous immunosuppressive oligodeoxynucleotide) could induce systemic immunosuppression during the later stages of wound healing to aid wound healing [163]. For UCMSC-Exos, the best osteochondral repair results were obtained when 3D-Exos was combined with 3D-printed scaffolds (Fig. 6B) [164]. Just like 2D-EVs, we can further engineer 3D-EVs to enhance the effects of 3D-EVs by loading them with specific substances or stimulating the growth of SCs from which they are derived in vitro. This not only addresses the issue of limited EV quantity, but also further enhances their effects, thus contributing to the clinical translation of EVs.

Currently, the applications of EVs derived from 3D cultured BMMSCs and UCMSCs were the most reported, and EVs derived from 3D cultured SCs were the most common in the study of injured skin healing, angiogenesis and neuroprotection. The effect of EVs produced from the same SCs applied to different 3D culture methods, such as scaffold-free (e.g., 3D culture based on ultra-low adhesion plates) or with scaffolds (e.g., based on hydrogels, collagen scaffolds, 3D printed hydroxyapatite scaffolds, etc.), static (the above mentioned static cultures without scaffolds or with scaffolds) or dynamic (e.g., 3D spinner flask culture, 3D aggregation wave reactor, etc.), may be altered, which may be due to the fact that different 3D culture methods create different microenvironments for cells, resulting in changes in the relevant properties of the extracellular matrix such as stiffness, viscoelasticity and others [180–182], thus affecting the biological behavior of cells, resulting in alterations in the yield and characteristics of EVs. EVs derived from 3D cultured SCs have been examined in various types of injuries including in skin, liver, lung, kidney, brain, SCI, bone defect and AMI. However, further exploration of the potential benefits of these EVs is still required.

## Conclusions and perspectives

Although 3D culture can increase the yield of SC-EVs and improve their activity in some aspects to enhance their effects, different 3D culture methods result in different biophysical factors such as shear stress and mechanical structure [143], which have a significant impact on the properties of EVs and lead to the production of EVs with large differences. However, there are many different 3D culture methods at present, and even the EVs produced by the same culture method still need to be further characterized. While there are currently standards for the identification of EVs, the EVs derived from 3D cultured cells still require uniform standards to ensure standardization. This is because 3D culture is distinct from 2D culture, and different 3D culture methods lead to variations in EV characteristics, limiting the versatility of 3D culture and hindering its clinical application. In the future, common standards can be established for EVs produced by 3D cultured cells of the same method, such as a set of standards for 3D culture using the suspension droplet method, a set of standards for those using the scaffold method, and a set of standards for those using the bioreactor method. Furthermore, when comparing the effects of 3D-EVs with 2D-EVs, some studies used the same number of particles as a criterion, and some used the same protein concentration as a criterion, but the two are not completely unified, and a set of standard interventions, such as the simultaneous verification of the same number of particles and the same protein concentration, are also needed in the future.

Exogenous intervention in 3D culture may enhance the efficacy of EVs [147], so we can add some intervention factors in future studies and combine them with the disease models studied to enhance some functions of EVs and better generate and apply EVs. We can also engineer 3D-EVs, or combine 3D-EVs with biomaterials. According to our research, we can select appropriate 3D culture methods and appropriate biomaterials to further improve the applicability of EVs [164, 183], which will not only speed up experimental research, but also save manpower and material resources. Although it has been confirmed that the contents of 3D-EVs, including proteins and miRNAs [148], significantly differ from that of 2D-EVs, but the mechanism of action of 3D-EVs remains largely unknown, and we don't know exactly what substances in 3D-EVs play a role. Therefore, future studies that delve deeper into the differences between the two may be important in finding out what specific components in 3D-EVs play a role, and at the same time, it is helpful to find out the reasons why 3D culture changes EVs’ characteristics. The premise of these studies is that we need to verify the contents of 3D-EVs obtained each time to ensure consistency for quality control. In conclusion, there is still much progress needed for the clinical applications of EVs derived from 3D cultured SCs.

Furthermore, EVs derived from 3D culture of other cells has been progressively applied in research. Recent studies showed that EVs derived from 3D cultured dermal papilla cells exhibited significant advantages in promoting angiogenesis, accelerating wound healing and improving wound healing efficiency both in vivo and in vitro [184]. Consequently, researchers can select the corresponding cells for 3D culture according to the disease under study, so as to obtain EVs more suitable for this disease and achieve better therapeutic effects. This discussion on the existing application studies of EVs derived from 3D cultured SCs to provide a foundation for future expansion studies on EVs derived from 3D cultured other cells.

Although the research on EVs derived from 3D cultured SCs is exciting, there are many limitations to the clinical translation and application of EVs. One of the problems with EVs is the lack of standardized protocol for purification. Currently, there are many methods to isolate EVs, such as ultracentrifugation, immunoaffinity capture, size exclusion chromatography and others [185]. However, none of them is perfect and each method has its own advantages and disadvantages. Therefore, a standardized protocol to isolate and purify EVs for clinical use is urgently needed. The publication of minimal information for studies of extracellular vesicles (MISEV2023) [186] is important to push forward the standardization of EVs isolation and purification. Clinical applications require large quantities of EVs, and although the 3D culture method can increase the production of EVs, there is still a long way to go before the large-scale manufacturing of EVs. Moreover, EVs should be subjected to strict quality control before clinical applications, but there is a lack of documents on industry-standard quality specifications for EVs [187]. In addition, the safety evaluation of EVs for long-term in vivo application still needs to be tracked and recorded to determine whether EVs are safe and reliable in vivo. We believe that with the joint efforts of researchers around the world, the limitations on the applications of EVs will be solved step by step, and their applications in clinical diseases will be more promising and valuable.

## Data Availability

Not applicable.

## Abbreviations

SCs: Stem cells
EVs: Extracellular vesicles
2D: Two-dimensional
3D: Three-dimensional
MSCs: Mesenchymal stromal/stem cells
AdSCs: Adipose tissue-derived SCs
ESCs: Embryo-derived SCs
Exos: Exosomes
MVs: Microvesicles
SC-EVs: Stem cell-derived EVs
2D-EVs: EVs derived from 2D planar cultured SCs
3D-EVs: EVs derived from 3D cultured SCs
SCI: Spinal cord injury
iPSCs: Induced pluripotent stem cells
BMMSCs: Bone marrow MSCs
MSC-EVs: MSC-derived EVs
hucMSCs: Human umbilical cord MSCs
hucMSC-EVs: Human umbilical cord MSC-derived EVs
COPD: Chronic obstructive pulmonary disease
hBMMSCs: Human bone marrow MSCs
hBMMSC-EVs: Human bone marrow MSC-derived EVs
iPSC-EVs: Induced pluripotent stem cell-derived EVs
UCMSCs: Umbilical cord MSCs
ARDS: Acute respiratory distress syndrome
BPD: Bronchopulmonary dysplasia
UC: Ulcerative colitis
IBD: Inflammatory bowel diseases
RP: Retinitis pigmentosa
MHs: Macular holes
CD: Crohn’s disease
Exo-d-MAPPS: Exosome-derived multiple allogeneic protein paracrine signaling
CKD: Chronic kidney disease
BMMSC-EVs: Bone marrow MSC-derived EVs
UCMSC-Exos: Umbilical cord MSC-derived exosomes
hucMSC-Exos: Human umbilical cord MSC-derived exosomes
HPMSC-Exos: Human placental MSC-derived exosomes
HPMSC-EVs: Human placental MSC-derived EVs
MSC-Exos: MSC-derived exosomes
iPSC-Exos: Induced pluripotent stem cell-derived exosomes
hBMMSC-Exos: Human bone marrow MSC-derived exosomes
AdSC-Exos: Adipose tissue stem cell-derived exosomes
UCBMSC-EVs: Umbilical cord blood MSC-derived EVs
hESCs: Human embryonic SCs
PEG: Polyethylene glycol
PVA: Polyvinyl alcohol
PLGA: Poly lactic-co-glycolic acid
PLA: Polylactic acid
PCL: Polycaprolactone
2D-Exos: Exosomes derived from 2D planar cultured SCs
3D-Exos: Exosomes derived from 3D cultured SCs
GelMA: Gelatin methacryloyl
hBMMSC-MVs: Human bone marrow MSC-derived microvesicles
BMMSC-Exos: Bone marrow MSC-derived exosomes
UCBMSC-MVs: Umbilical cord blood MSC-derived microvesicles
hESC-Exos: Human embryonic stem cell-derived exosomes
hAMSC-Exos: Human amnion MSC-derived exosomes
hPDLSC-Exos: Human periodontal ligament stem cell-derived exosomes
hDPSC-Exos: Human dental pulp stem cell-derived exosomes
hDPSC-EVs: Human dental pulp stem cell-derived EVs
TBI: Traumatic brain injury
5XFAD: 5 Familial Alzheimer’s disease mutations
GFAP: Glial fibrillary acidic protein
DG: Dentate gyrus
AD: Alzheimer’s disease
HUVEC: Human umbilical vein endothelial cell
IBE: Ischemic brain extract
IBE-MVs: MVs secreted by IBE-stimulated hBMMSCs
VEGFs: Vascular endothelial growth factors
NGFs: Nerve growth factors
AKI: Acute kidney injury
AMI: Acute myocardial infarction
2D-MVs: Microvesicles derived from 2D planar cultured SCs
3D-MVs: Microvesicles derived from 3D cultured SCs
I/R: Ischemia/reperfusion
DSS: Dextran sulfate sodium
6-OHDA: 6-Hydroxy-dopamine
hPMSCs: Human placental MSCs
hAMSCs: Human amnion MSCs
hPDLSCs: Human periodontal ligament SCs
hDPSCs: Human dental pulp SCs
